# Pinostrobin: An Adipogenic Suppressor from Fingerroot (*Boesenbergia rotunda*) and Its Possible Mechanisms

**DOI:** 10.3390/foods11193024

**Published:** 2022-09-29

**Authors:** Htoo Tint San, Hnin Ei Ei Khine, Boonchoo Sritularak, Eakachai Prompetchara, Chatchai Chaotham, Chun-Tao Che, Kittisak Likhitwitayawuid

**Affiliations:** 1 Department of Pharmacognosy and Pharmaceutical Botany, Faculty of Pharmaceutical Sciences, Chulalongkorn University, Bangkok 10330, Thailand; 2 Department of Biochemistry and Microbiology, Faculty of Pharmaceutical Sciences, Chulalongkorn University, Bangkok 10330, Thailand; 3 Center of Excellence in Natural Products for Ageing and Chronic Diseases, Faculty of Pharmaceutical Sciences, Chulalongkorn University, Bangkok 10330, Thailand; 4 Department of Laboratory Medicine, Faculty of Medicine, Chulalongkorn University, Bangkok 10330, Thailand; 5 Center of Excellence in Vaccine Research and Development (Chula Vaccine Research Center), Faculty of Medicine, Chulalongkorn University, Bangkok 10330, Thailand; 6 Center of Excellence in Cancer Cell and Molecular Biology, Faculty of Pharmaceutical Sciences, Chulalongkorn University, Bangkok 10330, Thailand; 7 Department of Pharmaceutical Sciences, College of Pharmacy, University of Illinois at Chicago, Chicago, IL 60612, USA

**Keywords:** adipogenesis, obesity, fingerroot, *Boesenbergia rotunda*, Akt, MAPK, triglyceride, glycerol

## Abstract

Obesity is a critical factor for chronic metabolic syndromes. The culinary plant fingerroot (*Boesenbergia rotunda*) has been reported for its anti-obesity activity. The anti-adipogenic effects of pandurantin A, a main component of fingerroot cultivated in Indonesia, have been studied. Nevertheless, the suppressive effect and related mechanisms of pinostrobin, a major constituent of Thai fingerroot, on adipogenesis have never been thoroughly investigated. This study aimed to evaluate the potential of pinostrobin to inhibit adipocyte differentiation. Culturing pre-adipocytes from both mouse (3T3-L1) and human (PCS-210-010) with pinostrobin at non-toxic concentrations (5−20 µM) for 48 h obviously hindered their differentiation into mature adipocyte as evidenced by reduced cellular lipid droplets. The lower levels of lipid metabolism-mediating proteins, namely C/EBPα, PPARγ, and SREBP-1c, as well as cellular triglyceride content were demonstrated in pinostrobin-treated 3T3-L1 cells when compared to the untreated control group. Additionally, pinostrobin modulated the signals of MAPK (p38 and JNK) and Akt (Akt/GSK3β, Akt/AMPKα-ACC). These findings suggest the benefit of fingerroot as a source of phytopharmaceuticals for obesity prevention and management, with pinostrobin as the active principle.

## 1. Introduction

Obesity is a serious global health problem responsible for 2.8 million premature deaths each year due to its close association with numerous diseases, such as type 2 diabetes, hypertension, atherosclerosis, cardiovascular disease, cancers, and mental concerns [1,2]. Individuals are considered overweight with a body mass index (BMI) ≥ 25 kg/m^2^ and obese with BMI ≥ 30 kg/m^2^ [2]. During the recent coronavirus disease 2019 (COVID-19) pandemic, high BMI has been found to be a factor of increased risk of hospitalization [3,4]. Although lifestyle modifications are the first-line management for obesity, pharmacotherapy and bariatric surgery are options to increase the quality of life. Some frequently prescribed anti-obesity drugs, such as orlistat, liraglutide, and lorcaserin, are not only expensive but also cause adverse effects. Orlistat and liraglutide can cause fatigue, dry mouth, cramps, and fecal incontinence [5,6]. The appetite suppressant lorcaserin was withdrawn from the United States market in 2020 due to the high risk of developing cancer [7]. Over the past decades, medicinal and culinary herbs have attracted much attention as a promising source of natural and safe anti-obesogenic agents [8,9]. Some are household herbs and spices, such as turmeric, chili, and lesser galangal, whilst others are medicinal botanicals, such as green tea, ginseng, and ginkgo. Secondary metabolites possessing distinct chemical scaffolds, including propionic acid derivatives, flavonoids, lignans, curcuminoids, phytosterols, and alkaloids, have been shown to be the active ingredients, working through different mechanisms of action [10,11,12,13].

As an important metabolic organ, fat tissue or adipose tissue plays a key role in energy homeostasis, and it is made up by two main types. The expansion of white adipose tissue (WAT) located at subcutaneous and visceral tissues can occur through hyperplasia (the result of a process called adipogenesis) and hypertrophy of the adipocytes, both of which cause weight gain and obesity. In contrast, brown adipose tissue (BAT) is responsible for thermogenesis, i.e., burning calories to generate body heat [14]. It has been shown that curcumin from turmeric could reduce WAT formation by suppressing the development of mature adipocyte via activating Wnt/β-catenin signaling and AMPK phosphorylation [15,16], while capsaicin from chili could stimulate the conversion of subcutaneous WAT into BAT by stimulating browning-specific genes and inhibiting adipocyte differentiation [17,18]. Although an imbalance of energy intake and energy expenditure is known to be the primary cause of excessive fat accumulation leading to weight gain and obesity, malfunction of adipocytes, especially dysregulation of adipogenesis, may also instigate this abnormality [19]. Adipogenesis is a multistep process of proliferation and differentiation of pre-adipocytes into mature adipocytes that eventually leads to the formation and expansion of adipose tissue. Adipocyte differentiation comprises four steps, including cell cycle arrest, mitotic clonal expansion (MCE), early differentiation, and terminal differentiation. Such complex processes are regulated by several signaling molecules and transcription factors such as peroxisome proliferator-activated receptor-gamma (PPARγ), CCAAT/enhancer-binding protein alpha (C/EBPα), and sterol response element-binding protein-1c (SREBP-1c) [20]. Inhibition of these biomolecules at various stages of the adipogenic pathways has become a key strategy to attenuate adipocyte maturation. Fingerroot (also known as Chinese keys and Chinese ginger) is a culinary herb widely used in preparing Asian cuisines in China, India, Indonesia, Thailand, Malaysia, and Myanmar [21,22]. In Indonesia, the roots are used to add flavor to porridge, whereas in Thailand, they are indispensable in peppery shrimp soup, believed to help boost breast milk supply in lactating women. *Boesenbergia rotunda* (L.) Mansf. is the accepted scientific name for fingerroot [23]; however, in the literature, the plant is frequently referred to as *Boesenbergia pandurata* (Roxb.) Schltr [22,24]. Studies have shown that *Boesenbergia rotunda* (*B. pandurata*) produces flavonoids of various types, with a wide range of biological activities, such as antifungal, antibacterial, antiviral, anti-inflammatory, anti-cancer, anti-osteoporosis, antioxidant, and anti-obesity activities [22,24,25,26,27,28]. Interestingly, several dietary supplements for weight loss in the market indicate fingerroot as the main active ingredient [29]. Studies on high-fat-diet mice indicated that fingerroot extracts prepared from the materials collected from Indonesia could reduce body weight gain by decreasing the accumulation of visceral fat [26,30,31]. Pandurantin A was found to be the major component (0.8% *w/w* based on dried weight) and was shown to suppress adipogenesis in mouse pre-adipocytes via regulating AMP-activated protein kinase (AMPK) and PPARα/δ signals [26]. However, in our investigation of plant samples obtained in Thailand, pinostrobin was found to be the main constituent, accounting for about 1.2% *w*/*w* [32], in agreement with an earlier report [33]. This chemical discrepancy may stem from several possible causes, such as genetic variations, geographical dissimilarities, and other factors, which remain to be investigated. More importantly, our preliminary study in 3T3-L1 pre-adipocytes found that panduratin A was about four times more toxic than pinostrobin (see Section 3.1). Structurally, pinostrobin is a flavanone, whereas pandurantin A is a chalcone bearing a prenyl substituent. In this report, the inhibitory activity and related mechanism of pinostrobin on adipogenesis in mouse 3T3-L1 pre-adipocytes are addressed. The suppressive effect of pinostrobin on the differentiation of human PCS-210-010 pre-adipocytes is also reported.

## 2. Materials and Methods

### 2.1. Chemical Reagents

**Figure 1:**
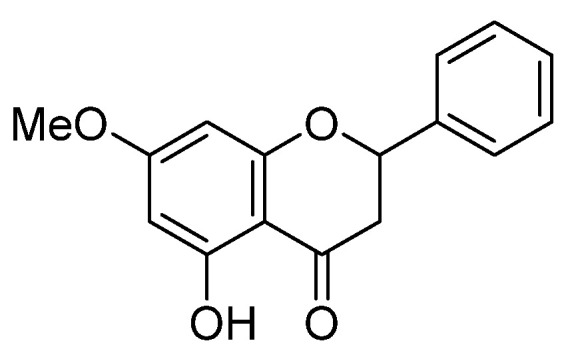
Chemical structure of pinostrobin.

The isolation and structural characterization of pinostrobin (>99% purity determined by NMR; melting point 96–97 °C) (Figure 1) from *B. rotunda* roots were carried out as previously described [32]. Oxyresveratrol (>98% purity) was purified from the heartwood of *Artocarpus lakoocha* Roxb. [34]. Crystal violet solution (1% *w*/*v*), formaldehyde solution (37% *w*/*v*), dimethyl sulfoxide (DMSO), skim milk powder, Hoechst33342, propidium iodide (PI), and oil red O solution (0.5% in isopropanol) were purchased from Sigma Aldrich (St. Louis, MO, USA). Fibroblast basal medium (FBM) was obtained from the American Type Culture Collection (ATCC, Manassas, VA, USA). Fetal bovine serum (FBS), Dulbecco’s modified Eagle medium (DMEM), penicillin/streptomycin solution (10,000 units /mL), l-glutamine (2 mmol/L), and trypsin (0.25%) were procured from Gibco (Gaithersburg, MA, USA). Insulin was purchased from Himedia (Mumbai, India). Isobutylmethylxanthine (IBMX), dexamethasone, bicinchoninic acid (BCA) protein assay kit, Western chemiluminescent ECL substrate, and radio-immunoprecipitation assay (RIPA) buffer were from Thermo-Fisher (Rockford, IL, USA). A protease inhibitor cocktail was obtained from Roche Applied Science (Indianapolis, IN, USA). Primary antibodies against GAPDH, Akt, p-Akt (Ser473), GSK3β, p-GSK3β (Ser9), AMPKα, p-AMPKα (Thr172), ACC, p-ACC (Ser79), PPARγ, C/EBPα, ERK1/2, p-ERK1/2 (Thr202/Tyr204), JNK, p-JNK (Thr183/Tyr185), p38, and p-p38 (Thr180/Tyr182) as well as horseradish peroxidase (HRP)-linked secondary antibodies were purchased from Cell Signaling Technology (Danvers, MA, USA). Primary antibody for SREBP-1c was from Thermo-Fisher (Rockford, IL, USA).

### 2.2. Cell Culture and Adipocyte Differentiation

Murine 3T3-L1 and human PCS-210-010 pre-adipocytes obtained from ATCC (Manassas, VA, USA) were, respectively, cultured in DMEM and FBM containing 10% FBS, 2 mmol/L l-glutamine, and 100 units/mL penicillin/streptomycin in humidified incubator at 37 °C with 5% CO_2_ and grown to 70−80% confluence before use. Cells between 5 and 17 passages were used in this study. 

For adipocyte differentiation, pre-adipocytes were seeded into a 24-well plate at a density of 2 × 10^4^ cells/well. After incubation for 48 h, the differentiation process was counted as day 0, and the cells were treated with differentiation medium containing 0.5 mM IBMX, 1 µM dexamethasone, and 10 µg/mL insulin with or without pinostrobin at non-toxic concentrations. On day 2 of differentiation, the medium was replaced with culture medium containing 10 µg/mL of insulin. On day 4, the cells were further incubated in culture medium with renewal every two days until lipid droplets were obvious upon microscopic examination, approximately on day 8 [35].

### 2.3. Cytotoxicity Assay 

Cytotoxic effect of pinostrobin in 3T3-L1 and PCS-210-010 pre-adipocytes was assessed by crystal violet colorimetric assay [36]. Briefly, after the cells (1 × 10^5^ cells/well in 96-well plate) were treated with 0−100 μM pinostrobin for 48 h, the detached dead cells were removed by washing 2 times with PBS (pH 7.4). The remaining living cells were fixed with 10% *w*/*v* formaldehyde for 15 min and stained with crystal violet (0.05% *w*/*v*) for 30 min at room temperature. Excessive crystal violet solution was removed via washing with deionized water for two times, and the 96-well plate was allowed to dry overnight. The stained cells were solubilized in methanol (100 μL) prior to absorbance measurement at 570 nm (A_570_) by a microplate reader (Anthros, Durham, NC, USA). The percent (%) cell viability was calculated on the basis of A_570_ ratio between pinostrobin-treated cells and the vehicle (0.5% DMSO)-treated control cells.

To evaluate cell death, Hoechst33342 and PI co-staining was performed with the cells incubated in the presence of pinostrobin for 48 h. The cells at a density of 1 × 10^5^ cells/well in 96-well plates were washed with PBS (pH 7.4), followed by 30 min incubation with 10 µg/mL of Hoechst33342 and 0.02 µg/mL of PI. The mode of cell death was determined by visual examination under an inverted fluorescence microscope (Olympus IX51, Olympus, Tokyo, Japan). Apoptotic cells were characterized by the bright-blue Hoechst33342 fluorescence of fragmented DNA and condensed nuclei. Necrotic cells were distinguished by the red propidium iodide fluorescence of DNA.

### 2.4. Assessment of Cellular Lipid Content 

Oil red O staining was used to determine the accumulation of cellular lipid droplets. After the differentiation process, the cells (2 × 10^4^ cells/well in 24-well plate) were washed with PBS (pH 7.4) and fixed with 10% *w*/*v* formalin for 15 min at room temperature. Then, oil red O solution was added to stain cellular lipid droplets for 1 h. After removal of excessive staining solution, the cells were rinsed three times with deionized water and 60% isopropanol. The stained adipocytes were observed under a Nikon Ts2 inverted microscope (Tokyo, Japan). The dye retained in the cells was extracted with 100% isopropanol, and the optical density (OD) was measured at 570 nm with a microplate reader (Anthros, Durham, NC, USA). The OD at 570 nm was calculated as a relative value compared to the total protein content (as determined by BCA assay kit) and presented as % oil red O staining.

In addition, the level of released glycerol in differentiated 3T3-L1 adipocytes was measured with a glycerol assay kit, and the amount of cellular triglyceride was determined with a triglyceride quantification kit, following manufacturer’s instructions (Sigma Aldrich, St. Louis, MO, USA). The triglyceride content was normalized with the total cellular protein content.

### 2.5. Cell Proliferation Assay 

Cell proliferation assay was performed to investigate the effect of pinostrobin on mitotic clonal expansion (MCE) during differentiation into adipocytes. After 3T3-L1 cells at a density of 1 × 10^5^ cells/well in 96-well plates were incubated for 2 days, the confluent cells were further cultured in differentiation medium with or without pinostrobin (0−20 μM) for 24, 48, and 72 h. Crystal violet staining was performed as described above to determine cell proliferation at each incubation time.

### 2.6. Western Blotting

The cell lysate was prepared from pre-adipocyte 3T3-L1 cells cultured in differentiation medium containing 0−20 μM pinostrobin for 48 h. Briefly, the cell membrane was broken by incubation with RIPA buffer supplemented with protease inhibitor cocktail on ice for 45 min. Equal amounts of protein samples as quantified by the BCA assay were separated through 10% SDS-PAGE and transferred electrophoretically onto nitrocellulose membranes (Bio-Rad Laboratories, Hercules, CA, USA). The nitrocellulose membranes were further blocked with 5% (*w*/*v*) skim milk in Tris-buffered saline containing 0.1% Tween 20 (TBST) for 1 h, followed by incubation with primary antibody overnight at 4 °C. The membranes were then washed with TBST (3 times × 5 min) and immersed in specific secondary antibody at room temperature for 2 h. Protein bands of interest were detected and quantified under UV light after reaction with western chemiluminescent ECL substrates using a chemiluminescence instrument (Chemiluminescent ImageQuant LAS 4000, GE Healthcare Bio-Sciences AB, Björkgatan, Uppsala, Sweden). The protein expression level relative to GAPDH (internal loading control) was calculated. 

### 2.7. Quantification of Gene Expression Using Real-Time Polymerase Chain Reaction (qRT-PCR)

Total mRNA was extracted from differentiated 3T3-L1 cells with Genzol reagent (Geneaid, Taiwan) according to the manufacturer’s recommended protocol. The cDNA synthesis kit (Thermo Scientific, Rockford, IL, USA) was used to synthesize single-stranded cDNA from 500 ng mRNA, which was quantified by a NanoDrop™ One/OneC Microvolume UV–Vis Spectrophotometer (Thermofisher Scientific, Rockford, IL, USA). The qRT-PCR reaction with the final reaction volume of 20 μL consisting of 10 μL of Bio-Rad Luna Universal qPCR master mix (Bio-Rad Laboratories, Hercules, CA, USA), 2 μL of 100 ng of cDNA template, 0.5 μL of each 10 μM forward and 10 μM reverse primers, and 7 μL of nuclease-free water (Thermo Scientific, Rockford, IL, USA) for volume adjustment was performed under the following thermal program: initial denaturation step at 95 °C for 3 min, followed by 40 cycles of denaturation at 95°C for 5 sec and primers annealing at 55 °C for 30 sec using the CFX 96 Real-Time PCR system (Bio-Rad Laboratories, Hercules, CA, USA). Gene expression levels were calculated by comparing the Cq values using the 2−ΔΔCq equation. The specific primers of PPARγ (forward: GATTCTCCTRTTGACCCAG, reverse: GARTGSGAGTGGTCTTCCAT), C/EBPα (forward: AGTCGGTGGACAAGAACAGC, reverse: GTGTCCAGTTCRCGGCTCA), SREBP-1c (forward: YTGCMGACCCTGGTGAGTG, reverse: ASCGGTAGCGCTTCTCAAT), and GAPDH (forward: 5′-GACCACAGTCCATGCCATCA, reverse: CCGTTCAGCTCAGGGATGAC) were obtained from Integrated DNA Technologies (Coralville, IA, USA). GAPDH was used as a housekeeping gene to normalize the differences in reverse transcription efficiencies.

### 2.8. Statistical Analysis

All experiments were performed as independent experiments in triplicate, and the results are presented as mean ± standard deviation (SD). Analysis of variance (ANOVA) was performed using the GraphPad Prism Version 7.00 for Windows (GraphPad Software, Inc., San Diego, CA, USA). A *p*-value < 0.05 was assumed as statistical significance.

## 3. Results

### 3.1. Suppressive Effect of Pinostrobin on Adipogenesis in 3T3-L1 Pre-Adipocytes

**Figure 2:**
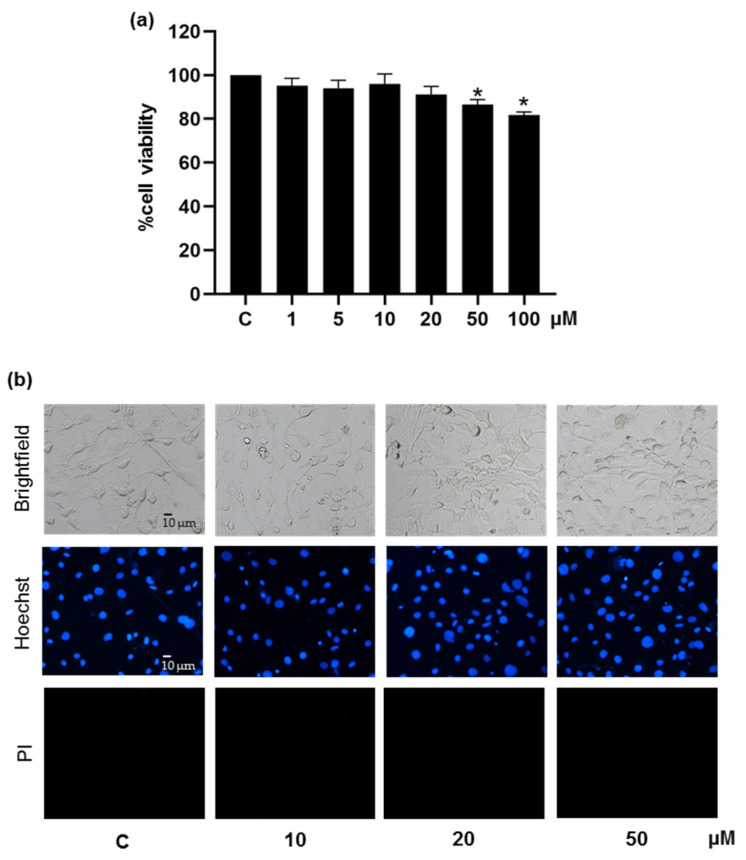
Cytotoxicity of pinostrobin in mouse 3T3-L1 pre-adipocytes accessed via (**a**) crystal violet viability assay and (**b**) Hoechst33342 (Hoechst) and propidium iodide (PI) co-staining for detecting mode of cell death. Data represents mean ± SD (n = 3). * *p* < 0.05 compared with vehicle control cells (C) treated with 0.5% DMSO.

Firstly, the range of non-toxic concentrations of the test compound was determined by crystal violet staining assay. Treatment with 1−20 μM pinostrobin for 48 h did not alter viability in pre-adipocyte 3T3-L1 cells as compared with the untreated control group (Figure 2a). On the contrary, panduratin A at concentrations higher than 5 μM significantly lowered cell viability (data not shown). Moreover, Hoechst33342 and PI co-staining provided evidence for the absence of toxicity of pinostrobin at 10−20 μM. As clearly seen in Figure 2b, bright blue Hoechst33342 fluorescence of condensed DNA/fragmented nuclei could be observed in cells cultured with pinostrobin at 50 μM. Noticeably, no necrosis was observed since no red PI fluorescence appeared in the treated cells. Therefore, the concentrations of pinostrobin from 1 to 20 μM were considered non-toxic and safe for use in subsequent experiments.

**Figure 3:**
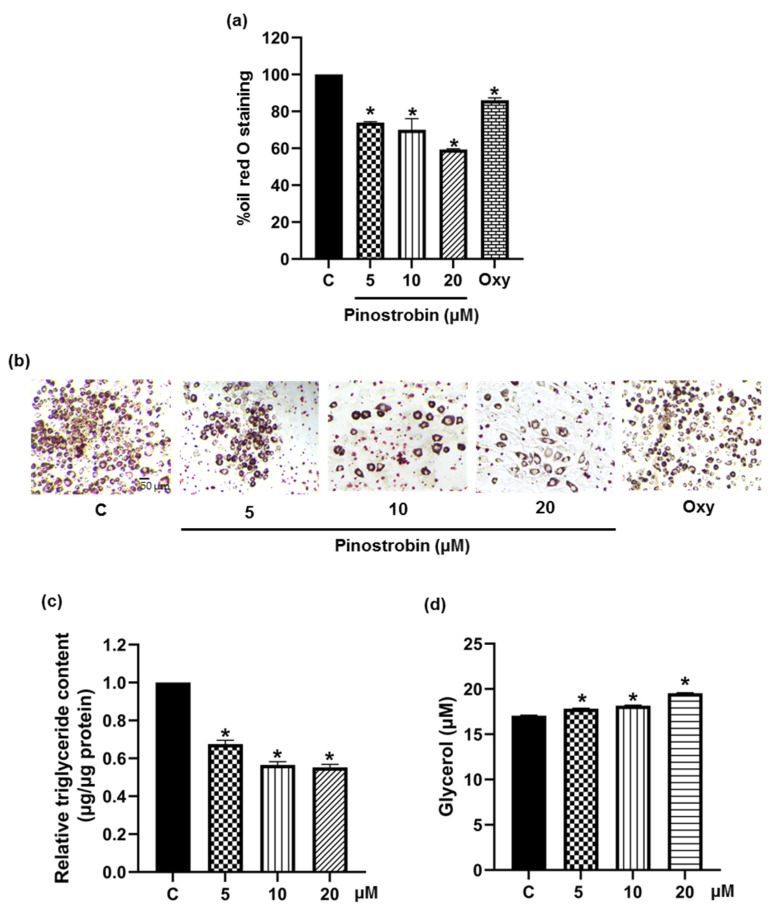
Suppressive effect of pinostrobin on adipogenesis was evidenced by the lower levels of (**a**) cellular lipid content presented as % oil red O and (**b**) cellular lipid droplets stained with oil red O in differentiated 3T3-L1 cells treated with 5–20 μM pinostrobin for 48 h. Oxyresveratrol (Oxy) at 5 μM was used as positive control. (**c**) A decrease of cellular triglyceride content and (**d**) an increase of extracellular glycerol level was detected in pinostrobin treated-3T3-L1 cells. Data represent mean ± SD (n = 3). * *p* < 0.05 compared with vehicle control cells (C) treated with 0.5% DMSO.

Intracellular lipid droplet accumulation was evaluated by oil red O staining after complete cell differentiation. Oxyresveratrol, a polyphenol earlier reported for anti-adipogenic activity in 3T3-L1 cells, was used as a positive control [37]. As demonstrated in Figure 3a, the culture incubated with differentiation medium containing 5−20 μM pinostrobin for 48 h significantly reduced intracellular lipid accumulation compared to the control cells. The ability of pinostrobin to decrease cellular lipid deposition was superior to that of oxyresveratrol when assessed at the same concentration of 5 μM (Figure 3b). Pinostrobin was further evaluated for its effects on cellular triglyceride and glycerol. Figure 3c,d indicates that pinostrobin at 5−20 μM significantly lowered the cellular triglyceride content and simultaneously increased the release of glycerol into the culture medium. These findings suggest the potential of pinostrobin as an adipogenic suppressor.

### 3.2. Effect of Pinostrobin on Cell Proliferation during Adipogenesis 

**Figure 4:**
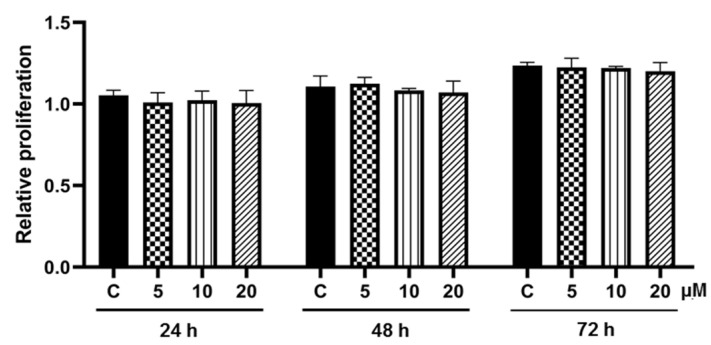
Effect of pinostrobin on mitotic clonal expansion in pre-adipocyte 3T3-L1 cells. Crystal violet assay was performed after 24–72 h cultured with differentiation medium with or without 5–20 μM pinostrobin. Cell proliferation was presented relative to the vehicle control cells (C) treated with 0.5% DMSO for 24 h.

Reentry into the cell cycle following rapid proliferation or mitotic clonal expansion (MCE) is an initial and crucial step for adipogenesis that happens in the first round at 24–36 h and the second round at 48–60 h during differentiation [38]. As depicted in Figure 4, a gradual increase in cell number was demonstrated in post-confluent 3T3-L1 cells cultured in differentiation medium for 24–72 h. Interestingly, there was no significant alteration in cell proliferation in cells co-treated with differentiation cocktail and pinostrobin (5–20 μM) when compared with the control cells. The results suggested that pinostrobin did not affect MCE during adipogenesis.

### 3.3. Inhibiton of the Expression of Adipogenic Transcription Factors by Pinostrobin

**Figure 5:**
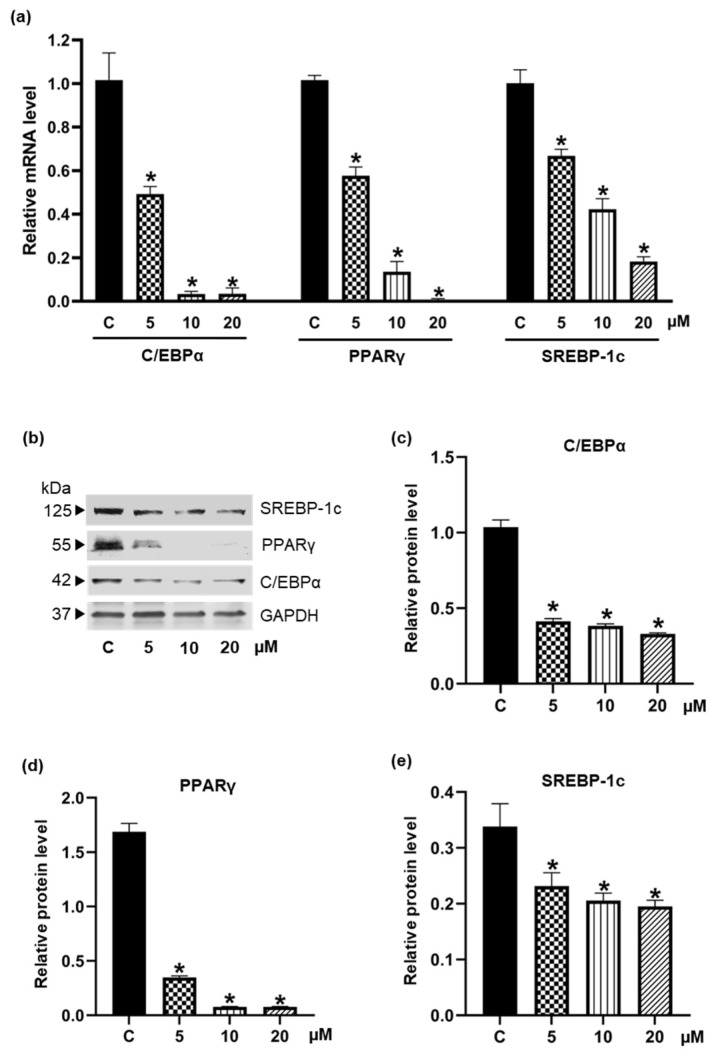
Diminution of adipogenic transcription factors in pinostrobin-treated 3T3-L1 cells. The expression of C/EBPα, PPARγ, and SREBP-1c was evaluated in the presence or absence of pinostrobin (5–20 μM) for 48 h via (**a**) qRT-PCR and (**b**–**e**) Western blot analysis. Data represent mean ± SD from three individual experiments. * *p* < 0.05 compared with vehicle control cells (C) treated with 0.5% DMSO.

Post-confluent 3T3-L1 cells were treated with 0–20 µM pinostrobin during the early differentiation step for 48 h, and the expression of adipogenic transcriptional factors was evaluated by qRT-PCR. Figure 5a indicates that pinostrobin decreased the mRNA levels of C/EBPα, PPARγ, and SREBP-1c in a concentration-dependent manner. In line with the diminished mRNA levels, protein levels of these transcription factors drastically reduced in the presence of 5–20 µM pinostrobin compared to the control group (Figure 5b−e). Images of the uncropped original Western blot are provided in Supplementary Figure S1.

### 3.4. Pinostrobin Downregulates Upstream Akt and MAPK Signaling Pathways

**Figure 6:**
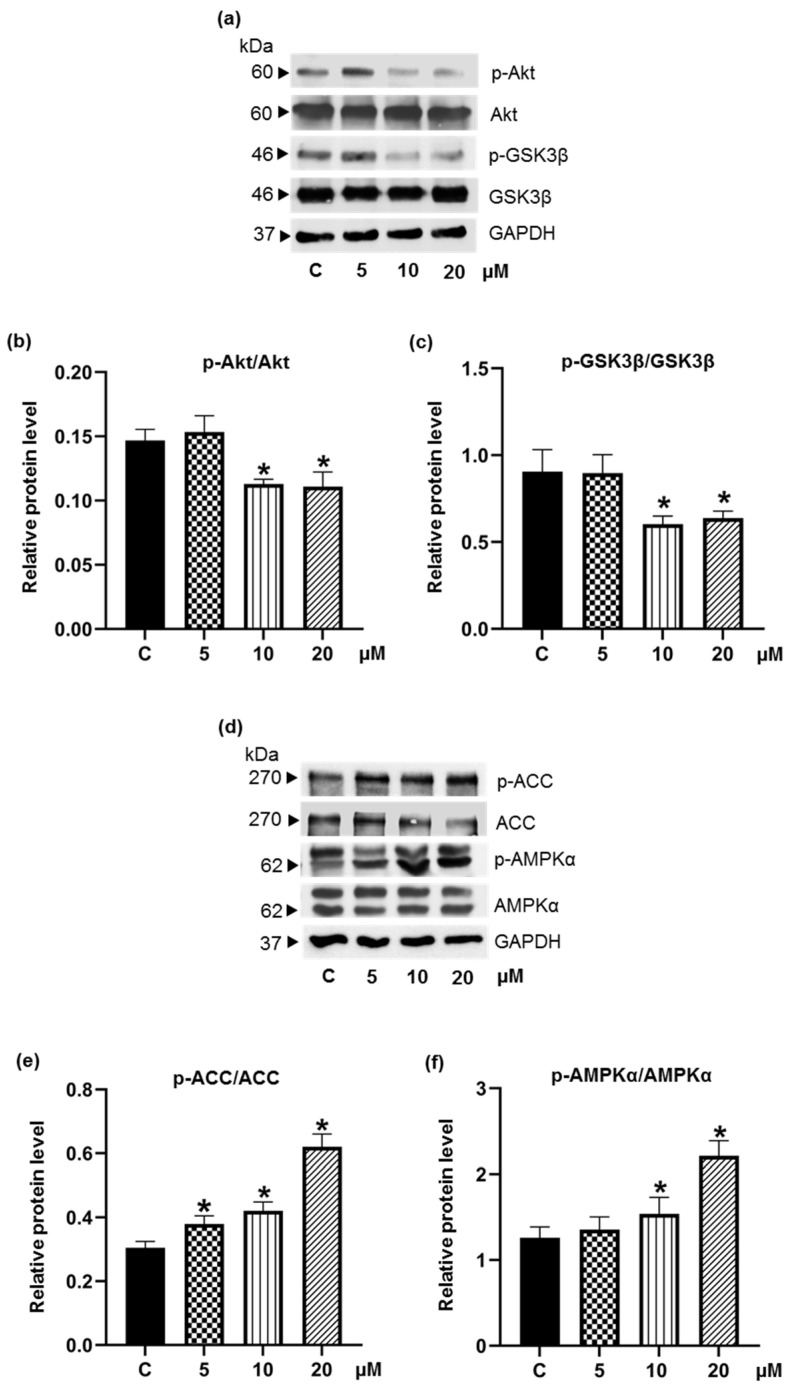
Effect of pinostrobin on the Akt-related signaling pathways. (**a**) Western blotting revealed the alteration of Akt and GSK3β signals in 3T3-L1 cells in the presence of pinostrobin for 48 h of the early differentiating process. There was significant reduction of (**b**) p-Akt/Akt and (**c**) p-GSK3β/GSK3β levels in cells treated with 10 and 20 μM pinostrobin. (**d**) The modulation on AMPK-ACC pathway, a downstream of Akt signal, was also detected in pinostrobin-treated 3T3-L1 cells. Activation of AMPK-ACC signal was evidenced by upregulated (**e**) p-AMPKα/AMPKα and (**f**) p-ACC/ACC levels. Data represent the mean ± SD. * *p* < 0.05 compared with vehicle control cells (C) treated with 0.5% DMSO.

The effects of pinostrobin on the upstream signaling pathways of adipogenesis in 3T3-L1 cells were further investigated. Serine/threonine protein kinase B (PKB or Akt) plays an important role in adipogenesis, and its activation enhances adipocyte differentiation. Activation of Akt (p-Akt) would upregulate SREBP-1c and promote lipogenesis [39]. Additionally, phosphorylated glycogen synthase kinase-3 beta (p-GSK3β) via Akt signaling would lead to the upregulation of C/EBPα transcription factor and promotion of adipocyte maturation [40]. In our hands, although no alteration of Akt and GSK3β levels was detected, a decrease in p-Akt expression with diminution of p-GSK3β was observed in cell cultures containing 10–20 μM pinostrobin (Figure 6a–c). Images of the uncropped original Western blot are provided in Supplementary Figure S2.

AMP-activated protein kinase (AMPK) serves as a key regulator in adipogenesis. Even though Akt and AMPK play different roles in the cellular metabolic process, they have common effects on the downstream transcription factor, SREBP-1c [41,42]. It is known that Akt negatively regulates AMPKα activity [43]. In agreement with the diminished p-Akt, 48 h incubation with pinostrobin (10–20 μM) stimulated the phosphorylation of AMPKα (Figure 6d,f). (Images of the uncropped original Western blot are provided in Supplementary Figure S3.) Surprisingly, as seen in Figure 6e, the p-ACC/ACC level was upregulated even at the lowest concentration (5 μM) of pinostrobin, which correlated well with the reduction of adipogenic transcription factors.

**Figure 7:**
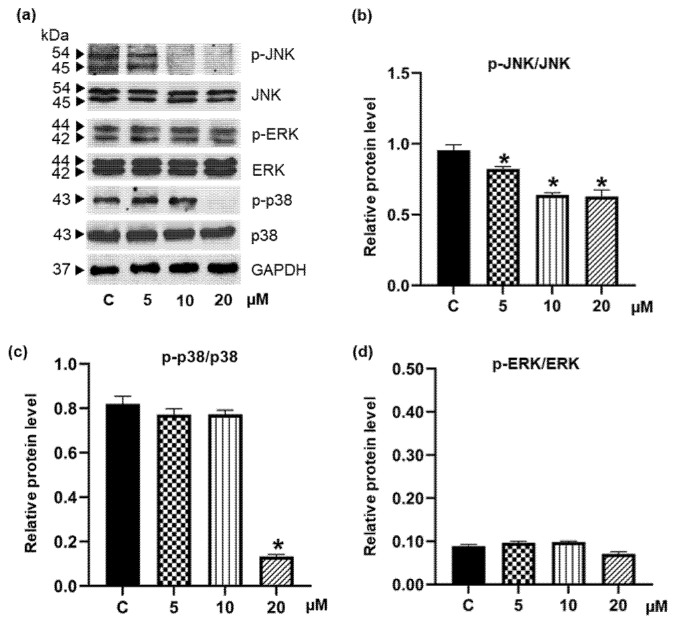
MAPK signals modulated by pinostrobin. (**a**) After induction into mature adipocyte for 48 h, the expression of MAPK signaling molecules, including JNK, ERK, and p38, was detected via Western blot analysis. (**b**) Treatment with low concentration of pinostrobin (5 μM) significantly downregulated the level of p-JNK/JNK, while (**c**) a dramatic decrease of p-p38/p38 was observed only in the presence of 20 μM pinostrobin. (**d**) Pinostrobin did not alter p-ERK/ERK levels in differentiated 3T3-L1 cells. Data represent the mean ± SD. * *p* < 0.05 compared with vehicle control cells (C) treated with 0.5% DMSO.

Several mitogen-activated protein kinases (MAPKs), including extracellular signal-regulated kinase (ERK), p38, and c-Jun N-terminal kinase (JNK), also play important roles during the maturation of adipocytes [44]. Suppression of these signaling molecules would effectively inhibit adipogenesis [45,46]. It was reported that inhibition of p38 hinders adipocyte differentiation via modulating PPARγ transcription. Our Western blotting analysis showed that pinostrobin significantly reduced the protein expressions of p-JNK and p-p38 (Figure 7a). (Images of the uncropped original Western blot are provided in Supplementary Figure S4.) Figure 7b depicts a dramatic decrease in the p-JNK/JNK level at a low concentration (5 μM) of pinostrobin as compared with the control group, while the p-p38/p38 level was repressed at higher concentrations (Figure 7c). It should be noted that there was no alteration of p-ERK/ERK levels in pinostrobin-treated 3T3-L1 cells (Figure 7d).

### 3.5. Pinostrobin Inhibits Adipocyte Maturation in Human Pre-Adipocytes

**Figure 8:**
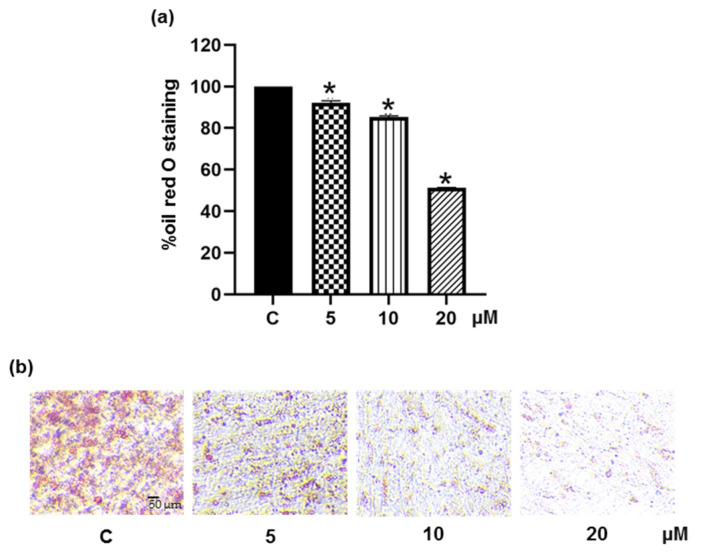
Suppressive effect of pinostrobin on adipogenic differentiation in human pre-adipocytes. Human PCS-210-010 pre-adipocytes were induced into matured adipocyte by culturing with differentiated medium in presence of 0–20 μM pinostrobin for early 48 h. After differentiation, cellular lipid was stained with oil red O solution for detecting (**a**) the lipid content representing as % oil red O and (**b**) accumulated lipid droplets observed under a microscope. Data represent the mean ± SD. * *p* < 0.05 compared with vehicle control cells (C) treated with 0.5% DMSO.

The anti-adipogenic potential of pinostrobin was further investigated in primary human PCS-210-010 pre-adipocytes. Cell differentiation was induced in the presence or absence of pinostrobin at non-toxic concentrations of 5–20 μM (data not shown), and the accumulation of cellular lipid droplets was assessed by oil red O staining assay. The cellular lipid content was found to decrease to 92.3%, 85.4%, and 51.2% upon treatment with pinostrobin at 5, 10, and 20 μM, respectively (Figure 8a). Figure 8b reveals a sharp reduction of cellular lipid droplets in pinostrobin-treated cells. These results suggest the ability of pinostrobin to suppress adipogenesis in human pre-adipocytes.

## 4. Discussion

In recent years, dietary interventions to manage overweight and obesity have gained tremendous attention, and the interest in using food as a therapeutic means is becoming popular [47,48]. Interest has also increased in the development of phytoconstituents for preventing and ameliorating obesity. Fingerroot, botanically known as *Boesenbergia rotunda* or *B. pandurata*, has been promoted for its beneficial potentials for weight loss. The extracts prepared from fingerroot samples from Indonesia attenuated diet-induced obesity in mice. The active principle was identified to be pandurantin A, a major compound that inhibited adipogenesis by modulating AMPKs and PPARα/δ signaling pathways [30]. However, from the fingerroot collected in Thailand, the main chemical component is pinostrobin, which, prior to this study, had no records of anti-adipogenic activity. In our preliminary examination, this compound possesses much less toxicity than panduratin A and thus warrants further investigation.

In the oil red O staining experiment in 3T3-L1 cells, pinostrobin reduced intracellular lipid storage with a magnitude greater than that of oxyresveratrol. As illustrated in Figure 3a,b, pinostrobin (5 μM) decreased lipid accumulation to 29.32 %, whereas only 13.93 % reduction was obtained for 5 μM oxyresveratrol treatment. Additionally, the reduced amount of cellular triglyceride and the elevated level of released glycerol confirmed that pinostrobin was able to inhibit the development of mature adipocytes (Figure 3c,d). It is worth noting that useful anti-obesogenic agents should not only suppress cell differentiation but also depress lipid accumulation in adipocytes [49,50]. The above findings suggest that pinostrobin could be a potential candidate.

The differentiation of pre-adipocytes into mature adipocytes requires the upregulation of several adipogenic regulating proteins, including PPARγ and C/EBPα. As the positive feedback loop, PPARγ activates the expression of C/EBPα, which is also necessary for stimulating PPARγ. The lipogenic transcription factors, sterol regulatory element binding proteins (SREBPs), also regulate the transcription of PPARγ [51,52]. Moreover, Payne et al. reported that C/EBPα regulates the expression of SREBP-1c, and decreased C/EBPα expression significantly reduces the transcription factor PPARγ [53]. In this study, treatment with pinostrobin decreased both mRNA and protein levels of these key adipogenic regulators at the early stage (48 h) of adipocyte differentiation (Figure 5). It has been reported that SREBP-1c would trigger the fatty acid synthase complex, resulting in the synthesis of triglyceride [54]. Furthermore, gene expressions responsible for lipid storage and insulin sensitivity are also modulated by C/EBPα [53]. These molecular pathways may account for the effects of pinostrobin observed in this study on the adipogenesis and cellular lipid metabolism. In passing, it should be mentioned that pinostrobin chalcone, a flavonoid structurally related to pinostrobin, possesses anti-adipogenic activity in mouse C3H10T1/2 adipocytes by suppressing the downstream signaling related to PPARγ, C/EBPα, and fatty acid-binding protein 4 (FABP4) [55].

As the Akt and MAPK pathways critically mediate adipogenesis [20], the alterations of proteins related to these processes were studied in differentiated pre-adipocytes in the presence of pinostrobin. The restraint of p-Akt/Akt signal (Figure 6b), in association with the reduction of downstream p-GSK3β (Figure 6c) as well as PPARγ and C/EBPα transcription factors in pinostrobin-treated 3T3-L1 cells (Figure 5) conforms with the fact that Akt/GSK3β cascade is essential for the expression of CCAAT-enhancer binding family proteins (C/EBPβ, C/EBPα) and PPARγ during adipocyte differentiation [56]. It is well-established that AMPK, which acts as a sensor for energy homeostasis by regulating several metabolic pathways, is negatively controlled by Akt signaling [39,40,41]. The phosphorylated form or active AMPK directly or indirectly suppresses the transcription factors for both adipogenesis and lipogenesis, resulting in cellular energy expenditure and suppression of synthesis of triglycerides and fatty acids [57]. The α1 subunit of AMPK is considered as a key subunit in adipose tissue [58,59]. The hinderance of phosphorylation at Thr172 of AMPKα sufficiently increases adipogenesis in 3T3-L1 pre-adipocyte [60,61]. Moreover, siRNA-down-regulated AMPK enhances lipid accumulation via mediating ACC consequence with the upregulation of C/EBPα/β, PPARγ and SREBP-1c in both mouse and human mesenchymal cells [62]. Consistent with several studies indicating that several flavonoids could suppress adipogenesis through modulation of the AMPK pathway [30], an increase in the levels of p-AMPKα/AMPKα and p-ACC/ACC (Figure 6e–f), together with lowered p-Akt/Akt ratios (Figure 6b), was observed in pinostrobin-treated pre-adipocytes. 

It has been established that activation of AMPK hinders the synthesis of triglycerides and fatty acids by inhibiting fatty acid synthase (FAS), acetyl-CoA carboxylase 1 (ACC1), and SREBP-1c [42]. Moreover, SREBP-1c involves with the expression of lipogenic enzymes, including FAS and ACC1, which converts acetyl-CoA to malonyl-CoA [63]. It has also been reported that Akt can directly activate SREBP-1c by reducing the expression of the SREBP-1c inhibitor *Insig2a* [41]. Therefore, the effects of pinostrobin on the Akt-related cascade might be responsible for the inhibition of adipogenesis and triglyceride accumulation. Nevertheless, in this investigation, only pinostrobin at 10–20 µM could efficiently abolish p-Akt, an active form of Akt. Further investigation at a low concentration for other mechanisms is warranted before any conclusion can be drawn.

Fundamentally, the MAPKs, including ERK1/2, JNK, and p38, are involved in adipocyte proliferation and differentiation. The phosphorylation of ERK1/2, JNK, and p38 can enhance the activation of C/EBPα and PPARγ [64]. It was reported that phosphorylation of p38 and JNK is essential for adipocyte differentiation and that JNK and p38 inhibitors can reduce lipid accumulation in adipocytes [45]. Interestingly, pinostrobin at 5 µM promptly hindered the activation of JNK (Figure 7b), which was well-correlated with the suppressive effect on adipogenesis and cellular lipid storage though the repression of p-p38/p38 was observed only at 20 µM pinostrobin (Figure 7c). On the other hand, there was no alteration of p-ERK/ERK levels in differentiated 3T3-L1 cells cultured with pinostrobin at all concentrations (Figure 7d). ERK signaling is involved in rapid proliferation during the MCE process and the PPARγ expression [38,46]. The lack of modulatory effects of pinostrobin on the proliferation of post-confluent pre-adipocytes during adipogenesis might be due to the non-significant change of p-ERK levels. Thus, it seems that pinostrobin works on the MAPK signaling pathways, i.e., JNK and p38, but not on ERK.

The antiadipogenic activity of pinostrobin was also demonstrated in human pre-adipocytes [65,66]. Pinostrobin was found to suppress adipogenic differentiation in a dose-dependent fashion (Figure 8). Cellular lipid accumulation was significantly reduced to 50% by 20 μM pinostrobin.

## 5. Conclusions

**Figure 9:**
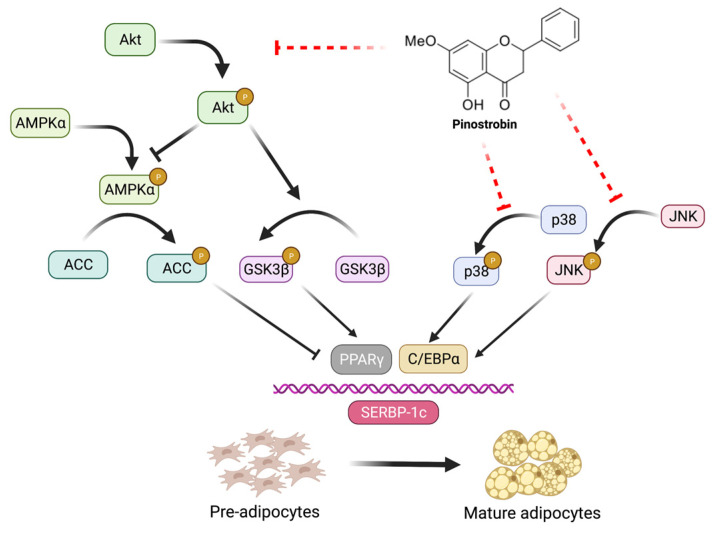
Proposed regulatory mechanisms of pinostrobin on the suppression of adipogenesis. The diminution of cellular lipid droplets and adipogenic transcription factors (PPARγ, C/EBPα, and SREBP-1c) in pinostrobin-treated adipocytes might be mediated through modulating Akt (Akt/GSK3β and Akt/AMPK-ACC) and MAPK (JNK and p38) signals. This figure was created with BioRender.com.

In summary, this study revealed for the first time about the suppressive effect of pinostrobin, the major flavonoid found in fingerroot (*B. rotunda*), on adipogenesis and its possible regulating machineries. Its regulatory role on Akt (Akt/GSK3β, Akt/AMPK-ACC) and MAPK (JNK, p38), in association with downregulated levels of PPARγ, C/EBPα, and SREBP-1c transcription factors, results in the suppression of adipogenesis and cellular lipid accumulation in differentiated adipocytes (Figure 9). The evidence obtained from this investigation supports the anti-obesogenic activity of fingerroot.

Pinostrobin, once established as an adipogenic suppressor, could be employed as a single ingredient or formulated with other herbs in food products for obesity prevention and management. However, pharmacokinetics, bioavailability, and toxicity studies in *in vivo* models are required before any clinical applications can be realized.

## Data Availability

Data are contained within the article.

## Supplementary Materials

The following supporting information can be downloaded at: www.mdpi.com/article/10.3390/foods11193024/s1, Figure S1: Original western blot images for Figure 5b: Suppression of adipogenic transcription factors in 3T3-L1 cells by 5, 10, and 20 µM pinostrobin; Figure S2: Original western blot images for Figure 6a: Effects of pinostrobin (5, 10, and 20 µM) on the Akt-related signaling pathway in 3T3-L1 cells; Figure S3: Original western blot images for Figure 6d: Effects of pinostrobin (5, 10, and 20 µM) on the AMPK-related signaling pathway in differentiated 3T3-L1 cells; Figure S4: Original western blot images for Figure 7a: Effects of pinostrobin (5, 10, and 20 µM) on the MAPK-related signaling pathway.
