# Supplementary material for: Pinostrobin: An Adipogenic Suppressor from Fingerroot (Boesenbergia rotunda) and Its Possible Mechanisms
Source: Foods. 2022 Sep 29;11(19):3024. doi: 10.3390/foods11193024 (PMC9564182; doi:10.3390/foods11193024)
Supplement: Supplementary file 1 [file foods-11-03024-s001.zip › foods-1872282-supplementary.pdf]

Supplementary Figures:

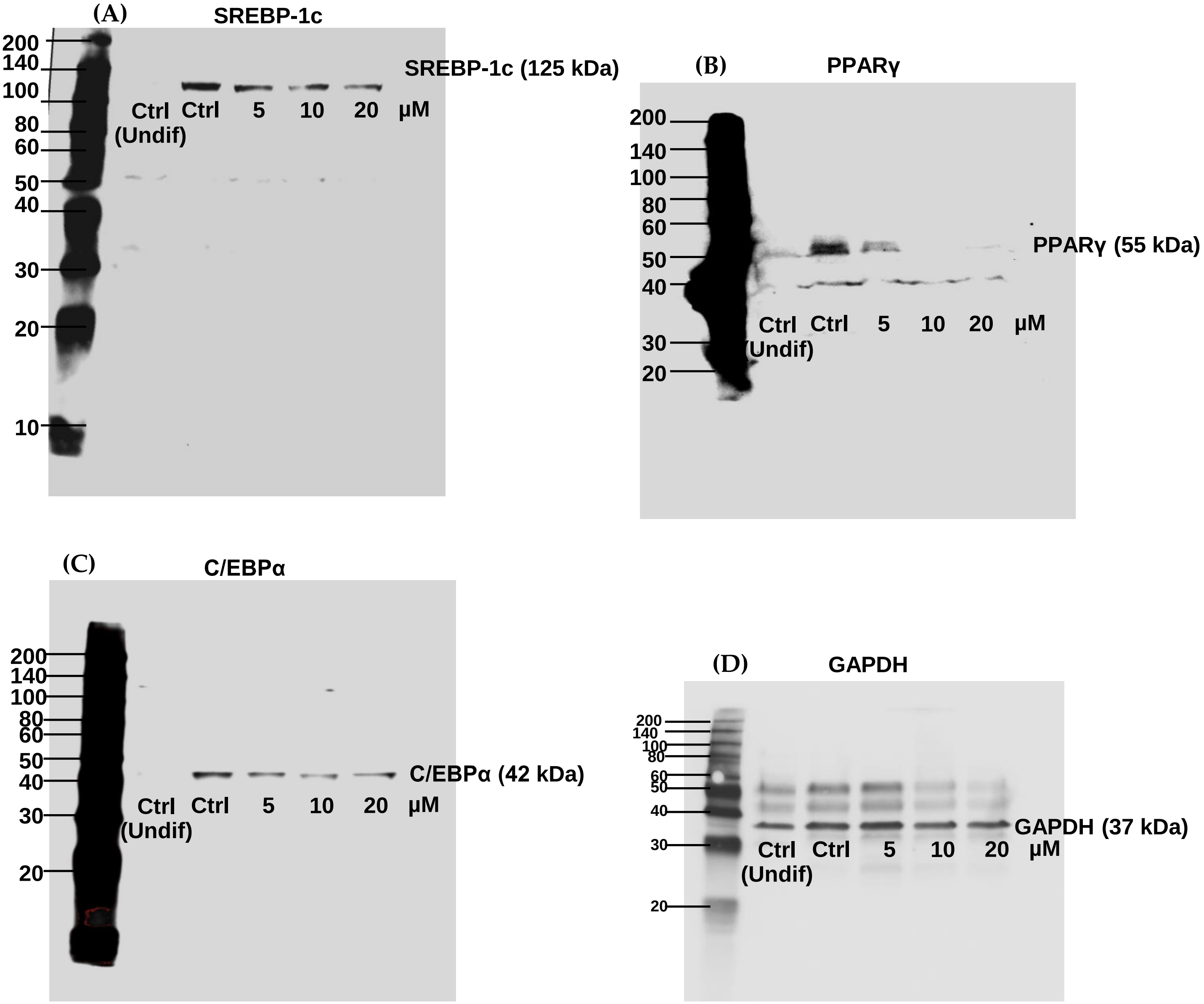

Ctrl (Undif): Undifferentiated control cells

Ctrl: Control cells

Figure S1. Original western blot images for Figure 5(b): Suppression of adipogenic transcription factors in 3T3-L1 cells by 5, 10, and 20 μM pinostrobin. Uncropped western blot images show molecular weight markers of (A) SREBP-1c (B) PPARγ (C) C/EBPα, and (D) loading control GAPDH. Significant suppressions were observed at all concentrations. For experimental details and statistical analysis, see the Experimental and Results sections.

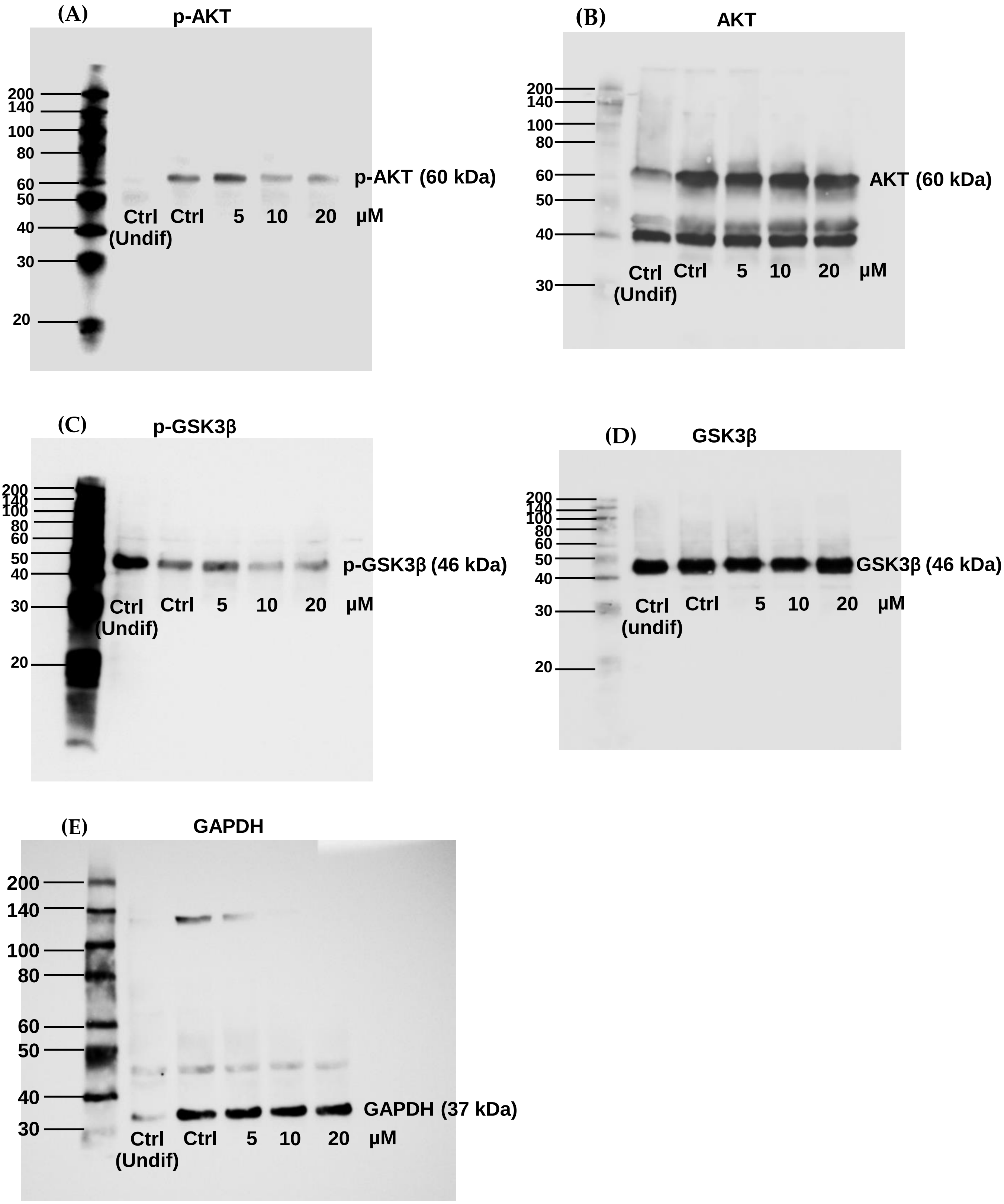

Ctrl (Undif): Undifferentiated control cells

Ctrl: Control cells

Figure S2. Original western blot images for Figure 6(a): Effects of pinostrobin (5, 10, and 20 μM) on the Akt-related signaling pathway in 3T3-L1 cells. Uncropped western blot images show molecular weight markers of (A) p-AKT (B) AKT (C) p-GSK3β (D) GSK3β, and (E) loading control GAPDH. A decrease in the p-Akt/Akt and p-GSK3β/GSK3β levels was observed at 10 and 20 μM but not at 5 μM pinostrobin. For experimental details and statistical analysis, see the Experimental and Results sections.

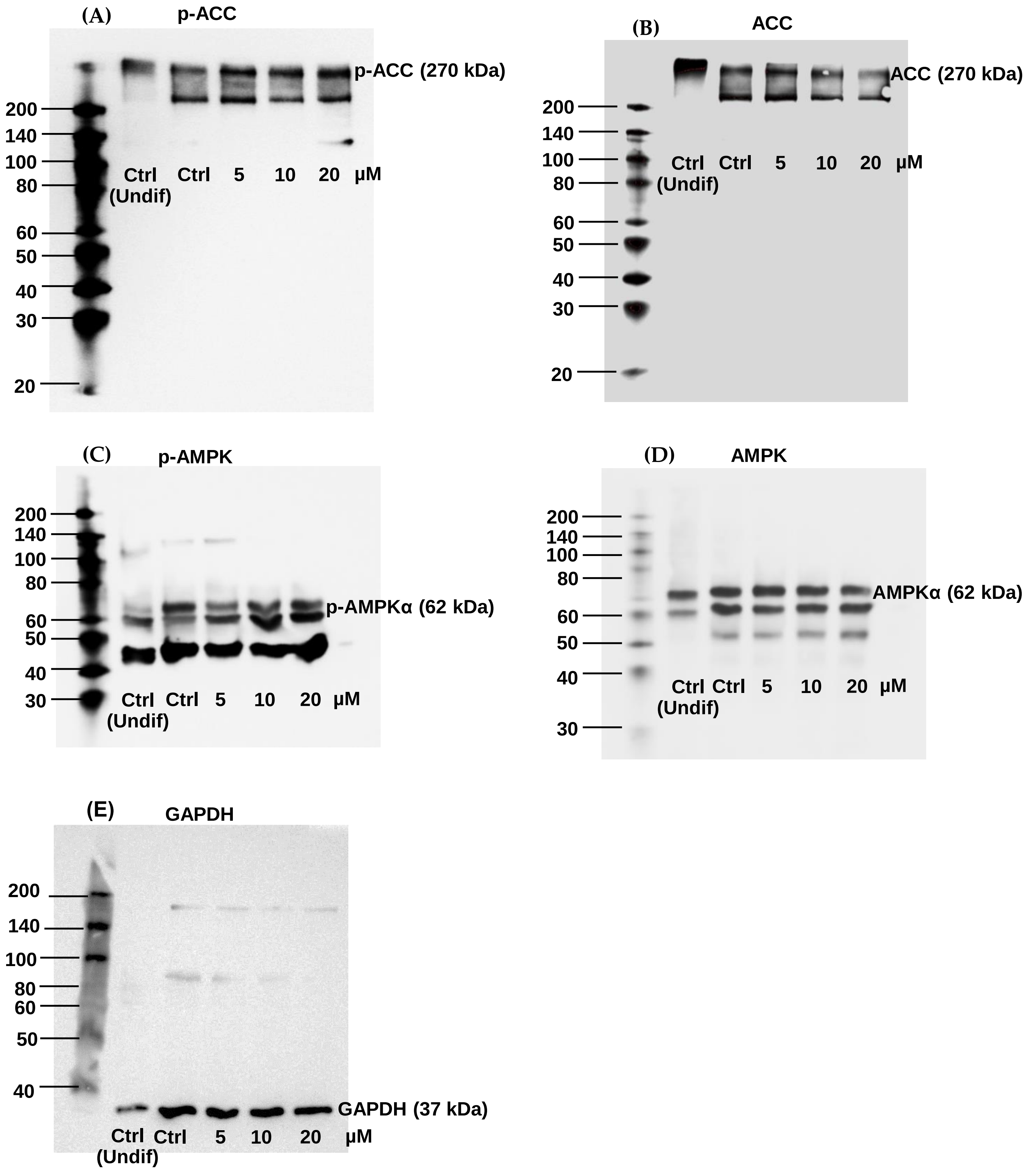

Ctrl (Undif): Undifferentiated control cells

Ctrl: Control cells

Figure S3. Original western blot images for Figure 6(d): Effects of pinostrobin (5, 10, and 20 μM) on the AMPK-related signaling pathway in differentiated 3T3-L1 cells. Uncropped western blot images show molecular weight markers of (A) p-ACC (B) ACC (C) p-AMPK*α* (D) AMPK*α*, and (E) loading control GAPDH. A significant increase in the p-ACC/ACC level was observed at all concentrations, but the upregulation of the p-AMPK*α*/AMPK*α* level was found only at the concentrations of 10 and 20 μM. For experimental details and statistical analysis, see the Experimental and Results sections.

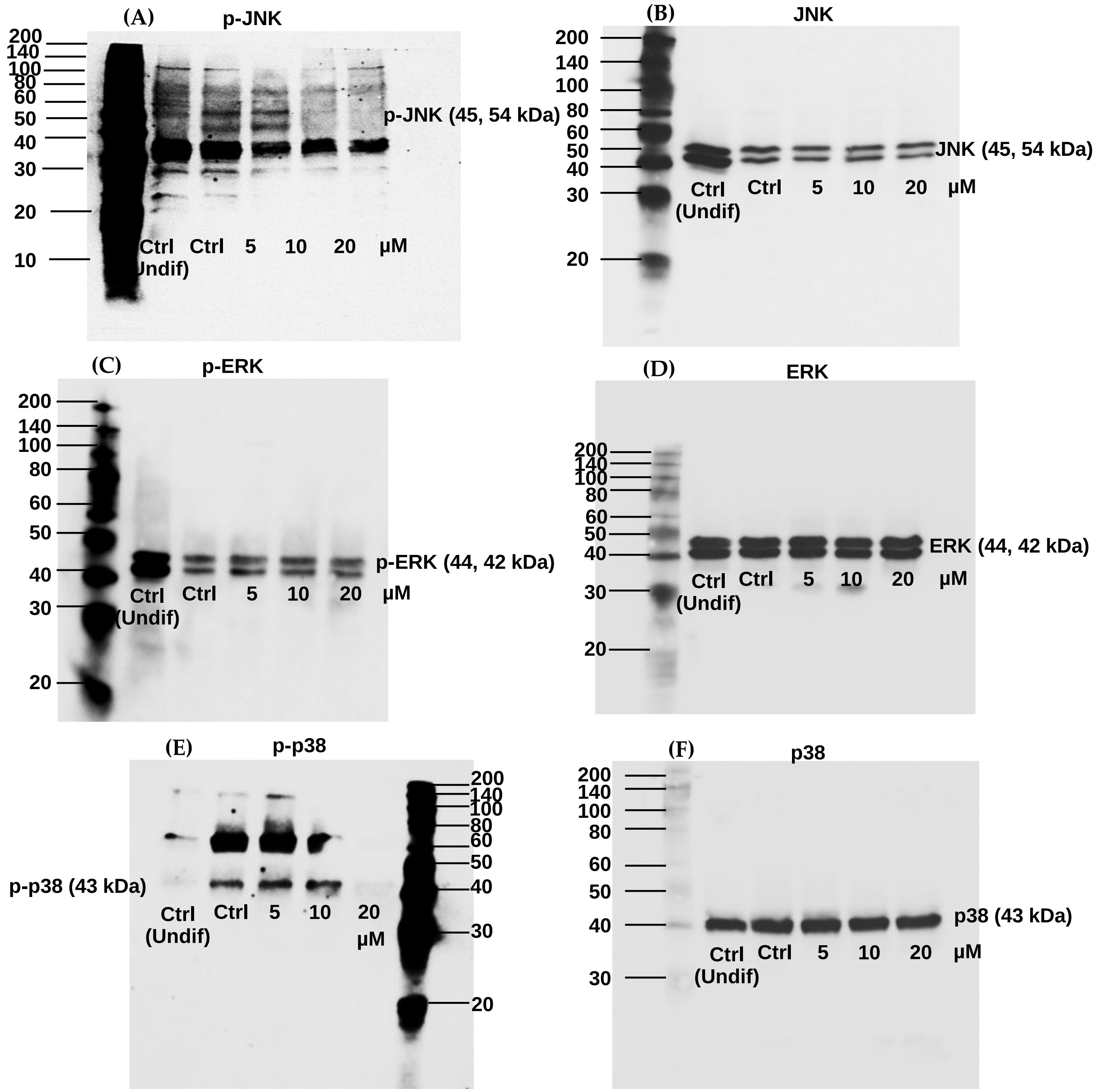

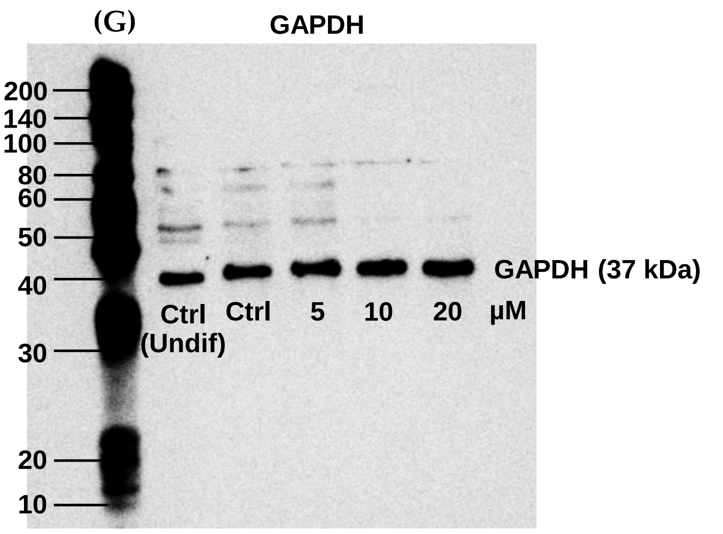

Figure S4. Original western blot images for Figure 7(a): Effects of pinostrobin (5, 10, and 20 µM) on the MAPK-related signaling pathway. Uncropped western blot images show molecular weight markers of (A) p-JNK (B) JNK (C) p-ERK (D) ERK (E) p-p38 (F) p38, and (G) loading control GAPDH. A significant decrease in the p-JNK/JNK level was observed at all concentrations, while the downregulation of the p-p38/p38 level was found only at a high concentration (20 µM). Pinostrobin at all concentrations showed no effects on the p-ERK/ERK level. For experimental details and statistical analysis, see the Experimental and Results sections.

Ctrl (Undif): Undifferentiated control cells

Ctrl: Control cells
